# Outpatient Subcutaneous Antimicrobial Therapy (OSCAT) as a Measure to Improve the Quality and Efficiency of Healthcare Delivery for Patients With Serious Bacterial Infections

**DOI:** 10.3389/fmed.2020.585658

**Published:** 2020-12-23

**Authors:** Tristan Ferry, Thomas P. Lodise, Jason C. Gallagher, Emmanuel Forestier, Sylvain Goutelle, Vincent H. Tam, John F. Mohr, Claire Roubaud-Baudron

**Affiliations:** ^1^Service des maladies infectieuses et tropicales, Hôpital de la Croix-Rousse, Hospices Civils de Lyon, Lyon, France; ^2^Université Claude Bernard Lyon 1, Lyon, France; ^3^Centre interrégional de référence pour la prise en charge des infections ostéo-articulaires complexes (CRIOAc Lyon), Hospices Civils de Lyon, Lyon, France; ^4^CIRI – Centre International de Recherche en Infectiologie, Inserm, U1111, Université Claude Bernard Lyon 1, CNRS, UMR5308, Ecole Normale Supérieure de Lyon, Univ Lyon, Lyon, France; ^5^Albany College of Pharmacy and Health Sciences, Albany, NY, United States; ^6^Department of Pharmacy Practice, Temple University, Philadelphia, PA, United States; ^7^Service de Maladies Infectieuses et Tropicales, Centre Hospitalier de Métropole Savoie, Chambéry, France; ^8^Pharmacie hospitalière, Hospices Civils de Lyon, Groupement Hospitalier Nord, Lyon, France; ^9^Univ Lyon, Université Lyon 1, UMR CNRS 5558, Laboratoire de Biométrie et Biologie Evolutive, Villeurbanne, France; ^10^Department of Pharmacy Practice and Translational Research, University of Houston, Houston, TX, United States; ^11^scPharmaceuticals, Burlington, MA, United States; ^12^CHU de Bordeaux, Pôle de Gérontologie Clinique, Bordeaux, France; ^13^Univ. Bordeaux, INSERM UMR 1053 BaRITOn, Bordeaux, France

**Keywords:** OPAT, OSCAT, BJI, healthcare system, antibiotics, catheter-related complications, subcutaneous antibiotic

## Abstract

Since the 1970s, outpatient parenteral antimicrobial therapy (OPAT) has been a viable option for patients who require intravenous antibiotics when hospitalization is not warranted. While the benefits of OPAT as a measure to improve the efficiency of healthcare delivery (i.e., reduced hospital days) and patient satisfaction are well-documented, OPAT is associated with a number of challenges, including line complications and reliance on daily healthcare interactions in some cases at home or in a clinic. To minimize the continued need for intensive healthcare services in the outpatient setting, there is trend toward patients self-administering antibiotics at home without the presence of healthcare workers, after adequate training. In most cases, patients administer the antibiotics through an established intravenous catheter. While this OPAT practice is becoming more accepted as a standard of care, the potential for line complications still exists. Outpatient subcutaneous antimicrobial therapy (OSCAT) has become an increasingly accepted alternative route of administration of antibiotics to IV by French infectious diseases physicians and geriatricians; however, currently, no antibiotics are approved to be administered subcutaneously. Antibiotics with longer half-lives that are completely absorbed and have a favorable local tolerability profile are ideal candidates for OSCAT and have the potential to maximize the quality and efficiency of parenteral antibiotic delivery in the outpatient setting. The increasing development of wearable, on-body subcutaneous delivery systems make OSCAT even more viable as they increase patient independence while avoiding line complications and potentially removing the need for direct healthcare professional observation.

## Introduction

Outpatient parenteral antimicrobial therapy (OPAT) is defined by the Infectious Disease Society of America (IDSA) as the administration of parenteral antimicrobial therapy in at least two doses on different days without intervening hospitalization. Dedicated guidelines for the prescription and management of OPAT have been published and updated in 2018 (1). OPAT is particularly relevant for the treatment of serious infections in patients who require long-term antibiotic therapy, especially when oral agents are not feasible, practical, or indicated, such as in bone and joint infections (BJI). However, OPAT has some drawbacks including the potential need for daily healthcare practitioner assessments due to the significant rate of catheter-related complications that can arise. To minimize the continued need for intensive healthcare services in the outpatient setting, there is a trend toward appropriate patients self-administering antibiotics at their own home, independent of healthcare workers. In most cases, patients administer antibiotics through an established intravenous (IV) catheter. However, the potential for IV catheter complications still exists with this practice, and there has been growing interest toward outpatient subcutaneous antimicrobial therapy (OSCAT) whereby the reliance on IV catheters can be eliminated. Herein, we (1) describe the limitation of current IV administration OPAT practices, (2) review available published data on SC administration of antibiotics in the outpatient setting, including PK data, (3) discuss the characteristics of parenteral antibiotics best suited for SC administration, and (4) review the potential use of wearable, on-body subcutaneous (SC) drug delivery systems that can be used to further facilitate the utility of OSCAT.

## Limitations Associated with Intravenous Administration of antibotics in the Outpatient Setting

Intravenous administration is the primary route used in OPAT. Every parenteral antibiotic can be administrated by this route, either by continuous, extended, or intermittent infusion. Some parenteral antibiotics are also approved for intramuscular injection. However, IM administration is impractical for longer courses of therapy due to pain. The treatment of BJI has been a common infection in which OPAT has been used as: (i) treatment courses often last several weeks; (ii) hospitalization is generally not needed; and (iii) oral agents may not be adequate (2).

Peripheral (midline) catheters or peripheral-inserted central catheters (PICC) have been extensively used for OPAT and are preferred due to their short-term use and lessened number of complications (1). They are mainly inserted through a radiological-guided procedure, simple to maintain, and easily removed. However, these types of intravenous access catheters have intrinsic disadvantages in this specific setting. Peripheral catheters must be changed every 4 days which is challenging for longer-term treatment, particularly in older patients who have poor venous network. Subclavicular or jugular central venous line exposes patients to unnecessary risks of infection and thrombosis and is prone to accidental withdrawal. Ports, while commonly used for outpatient administration of cancer chemotherapeutics, are not practical for patients who required parenteral antibiotics for a few weeks to months as their insertion and removal necessitate two surgical procedures.

Despite the clear advantages of midlines and PICC over other IV administration devices, they still associated with a number of potential complications (3). The most frequent is catheter occlusion which often requires an exchange of the catheter. The most concerning complications are IV catheter-related infections and thrombophlebitis (4, 5). In a systematic review of the literature, adverse event rates associated with vascular access devices ranged from 0 to 29% (6). In a single-center study evaluating 8,263 patients on OPAT over a 4-year period, 381 (4.6%) had at least one visit to the emergency department within 30 days of imitating OPAT and 104 ED visits (54% of OPAT-related ED visits and 27% of all ED visits) were due to occlusions and dislodgement of the intravenous catheter (7). Older patients are particularly vulnerable to experiencing complications during OPAT and influenced by the patient's cognition, mobility, and dexterity (1).

## Opat Trends and the Concept of Oscat in Clinical Practice

**Figure 1:**
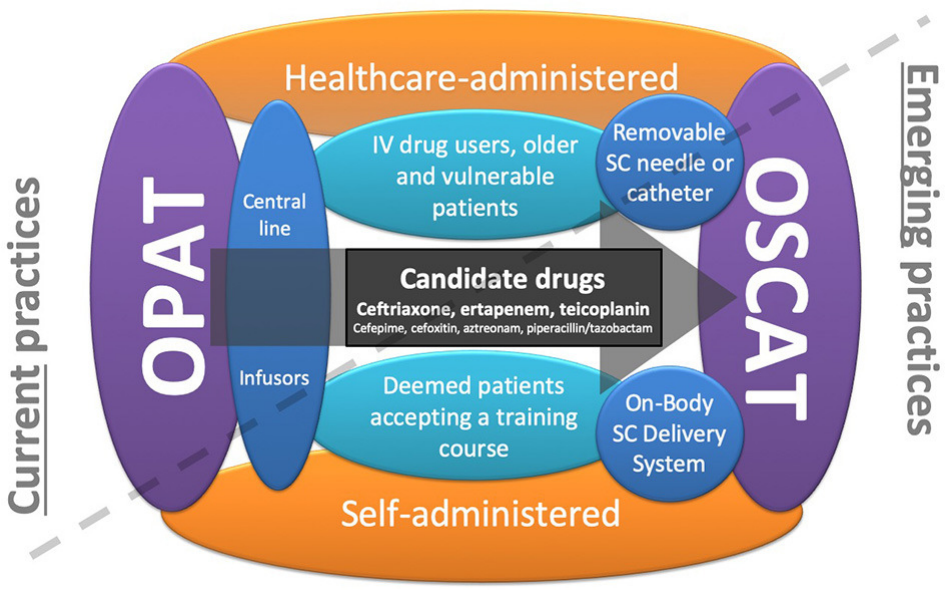
Current trends in outpatient parenteral antimicrobial therapy (OPAT): OPAT currently needs central lines and daily consumption of healthcare professionals at home. The first trend concerns self-administered OPAT that makes the patient more independent, and the second trend concerns the outpatient subcutaneous antimicrobial therapy (OSCAT) concept that avoids line complications. By cumulating these two trends, on-body subcutaneous delivery systems seem to be particularly relevant, making the patient more independent and avoiding line complications and the constraint of the daily passage of health professionals.

To date, OPAT has largely been delivered at physicians' offices/clinics or at patients' homes by home healthcare agencies. To minimize the continued need for intensive healthcare services in the outpatient setting, there are two main emerging practices in OPAT (Figure 1). The first trend is patient's self-administration of parenteral antibiotics after a training course independent of a home healthcare worker. Self-administration of IV antibiotics requires a degree of patient skill and responsibility and may not be practical for populations such as IV drug users, geriatric patients, and patients with cognitive or physical impairments. While this OPAT practice is becoming more commonplace, the potential for line complications still exists. In a study of 1,464 patients who received 1,950 OPAT courses at home, 9% of courses had at least one vascular access problem requiring clinical intervention. The most common complication was occlusion (49%), followed by accidental dislodgement (14%). Thrombosis and line infection occurred less frequently at rates of 0.34 and 0.16/1,000 OPAT days, respectively (8). However, recent studies of this method have not described increases in hospital readmissions nor complications in comparison with administration in the presence of a healthcare worker (3).

**Figure 2:**
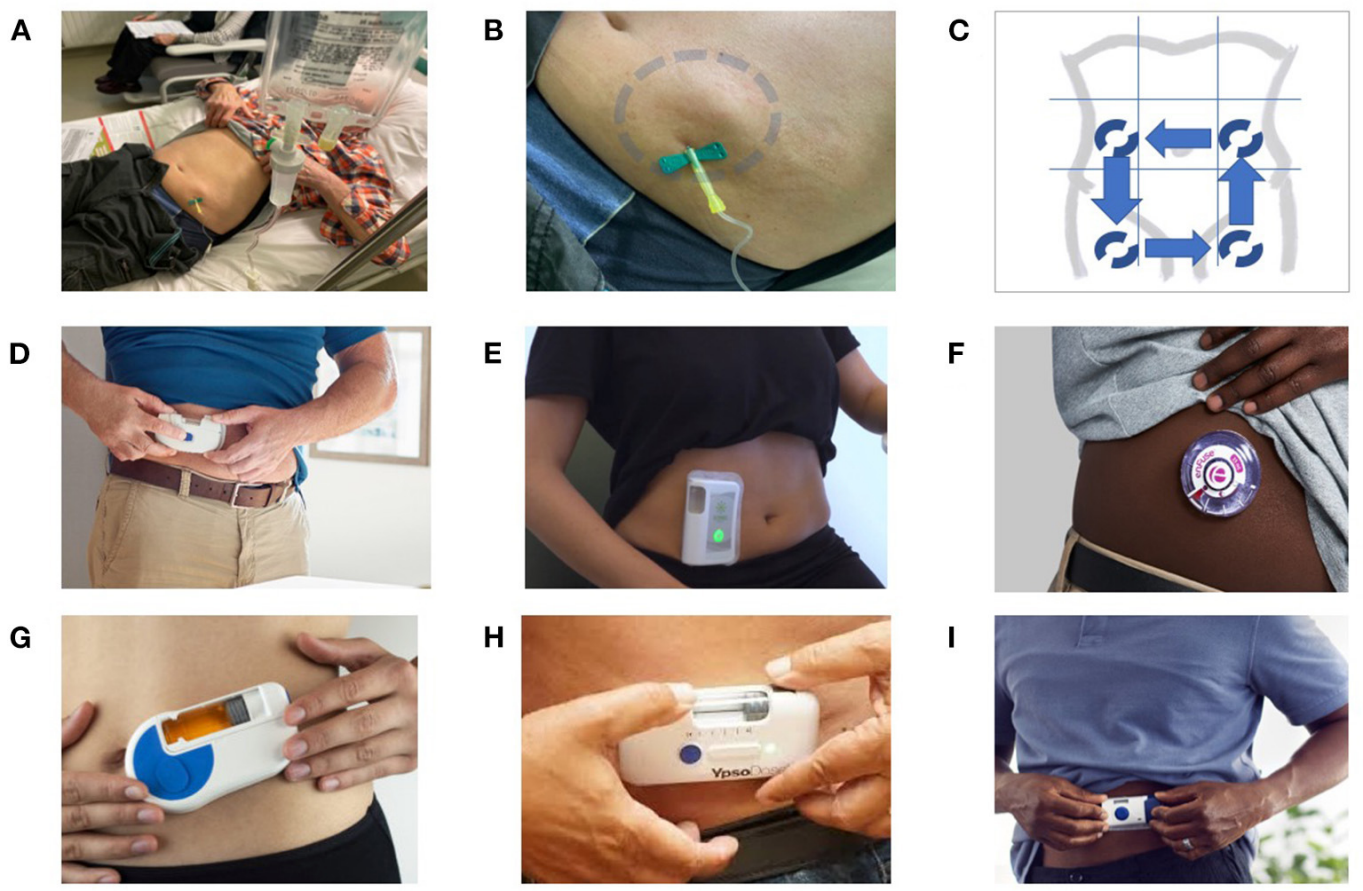
SC administration of antibiotics: from the French experience to the use of on-body delivery systems for subcutaneous drug delivery. **(A**–**C)** Example of an off-label SC administration in the French reference center for the management of complex BJI. An 80-year-old patient with a prosthetic joint infection required prolonged ertapenem therapy, as no oral options were available due to multidrug resistance, and the use of a catheter was considered to be not appropriate and feasible. As a consequence, instead of using a catheter for a daily single injection, the patient was treated with SC administration, with dilution of the drug in 50 ml of saline, and gravity infusion using a removable butterfly needle **(A)**. At the end of the 30–45-min injection, a tumefaction appeared around the injection site (blue circle), which gradually disappears over 15–30 min due to diffusion of the antibiotics **(B)**. Each injection was performed on different sites, with rotating alternation of the SC injections on the left flank, right flank, anterior face of the right thigh, and anterior face of the right thigh **(C)**. This patient, treated for several weeks, did not experience any local injection site adverse events, such as inflammation or necrosis. **(D–I)** Examples of on-body delivery systems for subcutaneous drug delivery: SmartDose® Gen II 10 ml (West Pharmaceuticals) **(D)**; Wearable on-body device utilizing a vial (Sorrel Medical) **(E)**; EnFuse® On-Body Infusor (Enable Injections) **(F)**; Wearable On-Body Large Volume Injector (Sonceboz) **(G)**; YpsoDose® (Ypsomed AG) **(H)**; and SmartDose® Gen I 3.5 ml (West Pharmaceuticals) **(F)**.

The second trend in OPAT is the use of SC administration. The major advantage of SC administration is that it minimizes the potential previously mentioned complications associated with IV catheter administration (9, 10). In addition, it is less demanding for nurses and could be performed at home or in long-term care facilities. There are a number of important considerations with the use of OSCAT as it is not yet FDA approved; however it is extensively used by French infectious diseases physicians and geriatricians (11) (Figures 2A–C). To facilitate OSCAT, there are now commercially available wearable, on-body SC delivery systems (Figures 2D–I). These devices make OSCAT more viable as they increase patient independence while avoiding line complications and remove the need for healthcare professionals. Below, we review the current published literature on SC administration of antibiotics in the outpatient setting, their PK properties by SC administration, identify the parenteral antibiotics best suited for SC administration, and review the potential use of wearable, on-body SC drug delivery systems that can be used to facilitate OSCAT.

## Subcutaneous Administration of Antibiotics: Current State of the Evidence

### Main Antibiotics

The first studies describing the use of SC administration of antibiotics in humans were published in the 1970s (12). In a large French national survey, based on voluntary participation, 367/382 (96%) of ID physicians and geriatricians reported prescribing antibiotics to be administered *via* the SC route (11). Of those surveyed who reported prescribing SC antibiotics, 100% reported to using ceftriaxone, they also prescribed teicoplanin, aminoglycosides, ertapenem, and amoxicillin in 39, 35, 33, and 15% of cases, respectively. In a retrospective study of 368 patients (mean age, 87 years) hospitalized in an acute geriatric unit of a Spanish public hospital treated with SC antibiotics between January 2012 and December 2016, ceftriaxone (233/368) and ertapenem (98/368) were the most commonly prescribed SC antibiotics (13). Case series report the use of SC route for administrating piperacillin-tazobactam (14), ceftazidime (15–17), and fosfomycin (18).

### Main Indications

The main reasons for utilizing the SC route were poor venous access, delirium, swallowing disorders, palliative care, tolerance, absence of oral active antibiotic drug, and facilitating hospital discharge or avoiding hospitalization (11, 19).

### Tolerance

In a prospective study evaluating the local tolerance of subcutaneously administered antibiotics in 219 patients (mean age, 83 years), 163 (74%) patients received ceftriaxone, 30 (13.7%) received ertapenem, and 10 (4.6%) received teicoplanin (19). Overall, 50 (22,8%) patients experienced 74 adverse events (AE) receiving ceftriaxone (*n* = 35/163), ertapenem (*n* = 7/30), and teicoplanin (*n* = 7/10). Pain was the most frequently reported local AE (*n* = 29). Other local AEs reported included hematoma (*n* = 16), induration (*n* = 17), and erythema (*n* = 6). However, no skin necrosis was reported. There was one AE considered to be severe, resulting in hospital readmission due to persistent induration and pain at the injection site; otherwise, AEs were transient. Reconstitution with lidocaine was used in ~30% of the cases and tended to decrease the occurrence of AEs (31% with lidocaine vs. 69% without) but not significantly (*p* = 0.09). Moreover, the use of a rigid catheter and a rapid infusion (<5 min) were associated with the occurrence of pain. In the abovementioned Spanish study, 3% of AE were reported (possibly underestimated due to the retrospective design of the study) mainly associated with aminoglycosides.

Considering all the SC ceftriaxone injections reported in the literature (~440 patients), only two cases of skin necrosis were reported and the most common AE was pain. With ertapenem (~200 patients), only one case of skin necrosis was reported. Local AEs have also been reported with teicoplanin (~110 patients); however, high concentrations were reported to have been administered. Skin necrosis was frequently described with SC admiration of aminoglycosides in several case reports (20–25).

### Main Infections

In a prospective evaluation of SC antibiotics conducted in France, the main sources of infection were urinary tract (44%), respiratory tract (33%), and BJI (7%). Several other publications from the same reference center for the management of complex BJI report the use of SC administration of antibiotics (26–28), especially, the authors described a prospective cohort of 10 patients (67–90 years) receiving SC prolonged suppressive antibiotic therapy for prosthetic joint infections or chronic osteomyelitis with a median treatment duration of 433 days, seven patients received SC ertapenem, three received SC ceftriaxone, and one received SC ceftazidime (one patient had sequential therapy with 8 days of ceftriaxone before switching to ertapenem). Six of the patients had a favorable outcome, for three patients, failure occurred after antibiotic cessation, one was lost to follow-up. One patient who received repeated direct injections of 2 g of ceftriaxone diluted to 2–5 ml final volume developed a skin necrosis (28).

### Main Pharmacokinetic Data

There is an increasing number of publications evaluating the pharmacokinetics (PK) of numerous antibiotics administered subcutaneously, particularly beta-lactams. In general, based on available literature, absorption of antibiotics after SC administration is complete resulting in bioavailability comparable with the same dose administered intravenously; however, the time to Cmax is prolonged and the overall Cmax is reduced (29).

Among the five studies evaluating PK of SC ceftriaxone (~80 subjects), three included healthy volunteers (30–32) and two were carried out in geriatrics and ID departments (33, 34). The average bioavailability of SC ceftriaxone across doses from 500 mg to 2 g was 96–107% compared with the IV route (30–32). The use of hyaluronidase before SC ceftriaxone injection increased the Cmax and shortened the time to Cmax (31).

Five studies collected PK data on SC ertapenem with patients (~60 patients) hospitalized in intensive care unit (35), ID (26, 27), or geriatrics (36), they confirmed a decreased Cmax and increased time to achieve it. After a 1 g dose of ertapenem, the bioavailability was 99 ± 18% after SC administration compared with IV (35). In a population PK analysis and PK/PD simulation based on the pharmacokinetics of IV and SC ertapenem in patients with BJI in a geriatric population, SC administration resulted in slightly higher or comparable time above the MIC compared with IV (27, 36).

Five studies reported SC teicoplanin PK data in patients (~80 patients) with suspected or confirmed nosocomial infections admitted to ICU, geriatric, or ID departments (37–41). AUC and Cmin were lower when teicoplanin was administrated with SC compared with IV during the loading phase; however, these differences disappeared overtime, indicating that IV route should be preferred during the first days of treatment and SC could be used afterwards with adequate Cmin and AUC/MIC (40).

In healthy volunteers, a SC infusion of cefepime or temocillin demonstrated comparable PK profile as an intramuscular or IV injection, respectively (42). Data on aminoglycosides PK (~60 patients) confirmed a decreased Cmax (12, 43–45).

## Candidate Antibiotics for SC Delivery

A suitable parenteral antibiotic candidate for SC administration is one that is absorbed completely, has a favorable PK/PD profile, and is well-tolerated. Due to the lower Cmax with SC administration, concentration-dependent antibacterials such as aminoglycosides are not good candidates; however, for time-dependent antibacterials such as beta-lactams, SC administration may be appropriate. A relatively long half-life is also a desired feature to enable OSCAT, as it may allow for less-frequent administration. With comparable PK profiles due to their complete absorption and longer half-lives, the pharmacodynamic profiles of ceftriaxone, ertapenem, and teicoplanin administered subcutaneously are comparable with those same profiles when the same antibiotic is administered intravenously making them ideal candidates from a PK and PK/PD perspective (27, 32, 37).

Another key consideration is local tolerability. The local tolerance of SC administration depends on several factors including the injection site, viscosity of the formulation, volume and rate of administration, pH, concentration, and osmolarity of the drug solution (46). In a study to evaluate local tolerability of SC administration of antibiotics, rapid infusion (<5 min), the use of a rigid catheter, and the class of antibiotic (teicoplanin) were significantly associated with a greater occurrence of AEs (19). However, during SC infusion of teicoplanin in patients with BJI, doses lower than 600 mg were better tolerated than higher doses (38). In a study evaluating the impact of injection volume on pain with SC injection, higher volumes were associated with increased pain (47). In a non-clinical study in Sprague-Dawley rats, tolerability of a SC infusion of ceftriaxone was concentration dependent and at high concentrations, no difference in tissue injury was observed between a bolus injection and a 2-h infusion (48). Based on the available literature, slower infusions of appropriate antibiotic concentrations could provide SC antibiotic therapies that are generally well-tolerated.

## Enabling Oscat With Wearable, on-Body Subcutaneous Drug Delivery Systems

With an increase in large-molecule drug development and the challenges of formulating a drug product that enables self-administration, there has been a surge in the development of wearable, on-body delivery systems. Nulasta® (pegfilgrastim, Amgen) and Repatha® (evolocumab, Amgen) are two examples of drugs that are administered subcutaneously *via* an on-body delivery system. There are many other systems that are in development that could be a potential solution to enable wider adoption of OSCAT (Figures 2D–I). Generally, these systems act as an infusion pump whereby they contain a medical-grade adhesive that is used to adhere the device to the skin of the arm, abdomen, or thigh after the drug is loaded into the system. Once placed, the device is activated, a needle is inserted into the SC space, and the drug delivery process begins. The rates of the infusions can be controlled either by pre-programmable electronics within the system, elastomeric tensions of drug reservoirs, or the inner diameter of the needle. Once the infusion is complete, the system is removed from the skin and discarded appropriately.

In order to ensure a particular on-body delivery system is appropriate for OSCAT, several considerations should be considered. First, since many parenteral antibiotics have limited stability after reconstitution, development of a novel, liquid formulation would be desirable. If this is not feasible, a specialized, simple, and patient-centric reconstitution system and method would be needed for patient self-administration. Second, the antibiotic must be compatible with the materials that it comes into contact within the on-body delivery system to ensure safety for the patient. Finally, the costs of the system should be appropriate to ensure that it is financially feasible for patients to be able to have access.

## Other Potential Trends for Outpatient Management

In addition to OSCAT, other innovative therapies have been developed to improve the management of serious bacterial infections in the outpatient setting. The use of oral antibiotics for some infections may offer an alternative to intravenous antibiotics in the outpatient setting. Recent randomized trials in patients with endocarditis (POET trial) and in BJI (OVIVA trial) demonstrated similar outcomes between treatment with oral antibiotics compared with intravenous antibiotics (49, 50). *In vitro* resistance could limit the utility of many oral antibiotics for infections caused by Gram-negative organisms; however, the availability of the oxazolidinones provide alternatives to intravenous routes for some serious infections caused by multidrug-resistant Gram-positive organisms (51). For serious bacterial skin infections requiring longer treatment durations, tedizolid may offer an advantage over linezolid due to the reduced potential to cause myelosuppression (52–54). In addition to oral antibiotics, the availability of long-acting lipoglycopeptides (e.g., dalbavancin and oritavancin) may also provide additional alternatives to OPAT as these drug half-lives ranged from 250 to 350 h (corresponding to 10–14 days), thus requiring only a few doses to provide a 4–6-week treatment course (55). As with OSCAT, additional data are required to further elucidate the utility of oral antibiotics and long-acting antibiotics in the management of serious bacterial infections where intravenous antibiotics have been recognized as a standard of care.

## Conclusions

In conclusion, OSCAT is an attractive alternative to the intravenous route of administration traditionally associated with OPAT. It has been used mainly in France primarily where prolonged courses are necessary and oral routes may not be feasible with some pathogens, such as in many BJI. Antibiotics with longer half-lives that are completely absorbed and are well-tolerated are ideal candidates for OSCAT. The concentration of the antibiotic, the osmolality of the solution, and the infusion rate contribute to the local tolerability of the SC infusion of the antibiotic. The availability of wearable, on-body SC drug delivery systems could improve the uptake of OSCAT while facilitating patient self-administration.

## Data Availability Statement

The original contributions presented in the study are included in the article/supplementary material, further inquiries can be directed to the corresponding author/s.

## Ethics Statement

Written informed consent was obtained from the individual(s) for the publication of any potentially identifiable images or data included in this article.

## Author Contributions

TF initiated the project and wrote the first draft of the manuscript. All authors participated in the literature review and the improvement of the manuscript.

## Conflict of Interest

TF received speaker honorarium from scPharmaceuticals. TL served as a consultant for scPharmaceuticals. JG served as an advisory board member for scPharmaceuticals. VT received honoraria and research grants from scPharmaceuticals. JM was SVP of Clinical Development and Medical Affairs for scPharmaceuticals who was developing antibiotics for subcutaneous administration. CR-B received research grant from the French Health authorities (*Agence Nationale de Sécurité du Médicament et des produits de santé*, ANSM) and SG from the *Foundation Innovations en Infectiologie* (FINOVI) to perform a prospective study about the pharmacokinetic and safety of subcutaneous antimicrobial therapy in France (*Pharmacocinétique et tolérance des antibiotiques administrés par voie sous-cutanée chez le patient âgé de plus de 65 ans*; PHASAGE study). The remaining authors declare that the research was conducted in the absence of any commercial or financial relationships that could be construed as a potential conflict of interest.
